# HBV-miR-3 and cGAS-STING axis: a new frontier in hepatitis B therapy

**DOI:** 10.3389/fcimb.2025.1662461

**Published:** 2025-08-06

**Authors:** Cao Hua, Saima Aftab, Zulqarnain Baloch

**Affiliations:** ^1^ Jiangxi Medical College, Shangrao, Jiangxi, China; ^2^ School of Automation Engineering, University of Electronic Science and Technology of China, Chengdu, China; ^3^ Biomedical Research Center, Northwest Minzu University, Lanzhou, China

**Keywords:** hepatitis B virus, cGAS-STING pathway, HBV-miR-3, immune evasion, viral persistence, antiviral therapy

HBV infection poses a significant global health burden, driving the development of liver cirrhosis and hepatocellular carcinoma (HCC) (Polaris Observatory, 2018). Despite progress in prevention and treatment, the mechanisms underlying chronic HBV infection and its persistence within the host remain incompletely understood. The interaction between HBV and the host immune system is crucial in shaping the infection’s progression (Riedl et al., 2021). Acute HBV infections can be resolved through strong immune responses, whereas chronic infections are defined by immune tolerance and sustained viral replication. HBV utilizes various strategies to evade immune detection, including interfering with antigen presentation. By disrupting the binding of major histocompatibility complex (MHC) molecules to antigenic peptides, HBV hinders the activation of adaptive immunity. Additionally, the virus produces proteins that suppress innate immune defenses, reducing cytokine production, T cell activation, and antibody responses. These mechanisms foster an environment that supports viral persistence and enables HBV to evade immune clearance, leading to chronic infection.

The cGAS-Stimulator of Interferon Genes (STING) pathway is a vital component of the innate immune response, responsible for detecting viral DNA and triggering the production of type I interferons (IFNs) (Amin et al., 2021; Zhao et al., 2023; Zheng et al., 2023). This pathway plays a pivotal role in recognizing cytosolic viral DNA, activating downstream signaling cascades that produce type I IFNs and other antiviral molecules to control viral replication and prevent the establishment of chronic infection. However, HBV employs sophisticated mechanisms to undermine this pathway (Amin et al., 2021; Zhao et al., 2023). For instance, the HBx mediates the ubiquitination of cGAS, effectively downregulating the pathway and diminishing the antiviral response. Additionally, HBV leverages HBV-miR-3, a microRNA encoded by the virus, to further suppress the cGAS-STING pathway, highlighting its ability to evade immune detection and promote viral persistence.

Recent research published in the World Journal of Hepatology has unveiled a pivotal role for HBV-miR-3 in undermining the host’s innate immune defenses (Zhen-Yu Xu et al., 2025). HBV-miR-3 directly targets the 3’-untranslated region (3’-UTR) of cGAS mRNA, leading to a reduction in cGAS protein levels through post-transcriptional regulation. This mechanism does not alter mRNA expression but effectively downregulates cGAS at the protein level, thereby impairing the functionality of the cGAS-STING pathway. A compromised pathway results in reduced STING phosphorylation and a subsequent decline in the production of type I IFNs, particularly IFN-β. This attenuation significantly weakens the host’s ability to mount an effective antiviral response, fostering an environment conducive to HBV persistence. Experimental models of HBV-infected hepatocytes have provided critical insights into the regulatory dynamics of HBV-miR-3. Studies have confirmed that HBV-miR-3 decreases cGAS protein expression without affecting mRNA levels, highlighting a post-transcriptional mechanism of immune modulation. This reduction in cGAS protein correlates with suppressed STING activation and a marked decline in IFN-β production, key components of the antiviral defense. Further investigations into the dose-dependent effects of HBV-miR-3 agomir—an enhancer of HBV-miR-3 activity—have shown a progressive suppression of cGAS expression and related immune responses. In contrast, the application of HBV-miR-3 antagomir—a molecule designed to inhibit HBV-miR-3—restores cGAS protein levels and reinvigorates the cGAS-STING pathway, thereby enhancing the innate immune response. Moreover, TRIM29 is shown to restrict antiviral innate immunity against DNA virus infections and viral myocarditis by targeting STING for degradation (Xing et al., 2017) (PMID: 29038422) and enhancing ROS-mediated TBK1 oxidation to inhibit type I IFNs production (Junying Wang et al., 2024). It has been reported that the expression of TRIM29 is controlled by non-coding RNAs, epigenetic modifications, and post-translational regulatory mechanisms (Qitong Wu and Sharma, 2024). It is possible that HBV-miR-3, by upregulating TRIM29 expression, could inhibit the cGAS-STING pathway, thereby contributing to immune evasion. TRIM29 has been shown to target STING for degradation and enhance ROS-mediated TBK1 oxidation, both of which prevent the activation of type I interferons and disrupt the antiviral immune response. By increasing TRIM29 levels, HBV-miR-3 may block the immune recognition of viral DNA, impair the host’s ability to mount an effective immune response, and promote chronic HBV infection. This suggests a mechanism by which HBV uses HBV-miR-3 to dampen innate immunity and evade host defenses.

The identification of HBV-miR-3 as a modulator of the cGAS-STING pathway offers promising therapeutic implications. Targeting HBV-miR-3 or enhancing the cGAS-STING pathway could restore innate immune responses and suppress viral replication. Potential strategies include the development of STING agonists, such as Schisandrin C, which have shown promise in activating the pathway and suppressing HBV replication. Additionally, antagomirs targeting HBV-miR-3 can counteract its inhibitory effects on cGAS, restoring immune function and complementing existing antiviral therapies.

Strengthening the host’s ability to detect and respond to HBV DNA may also improve viral clearance and reduce the risk of chronic infection. Despite these advances, several questions remain. Understanding how HBV-miR-3 expression is regulated during infection and its interplay with other immune pathways could provide a more comprehensive understanding of viral persistence. Translating findings from *in vitro* and preclinical studies into effective therapies for patients with chronic HBV infection remains a significant challenge.

The discovery that HBV can evade the immune system by manipulating the cGAS-STING pathway through HBV-miR-3 marks a significant advancement in our understanding of viral immune evasion. Given the central role of this pathway in innate immunity, it presents a promising therapeutic target. By exploring the molecular mechanisms that underlie HBV persistence, researchers can develop innovative treatments that not only suppress viral replication but also enhance the host’s immune response. Achieving these objectives will require collaborative efforts in basic research, translational studies, and clinical development, ultimately contributing to the global goal of eliminating HBV as a public health threat.
